# Considerations for the use of immunosuppression for the management of pemphigus during the COVID-19 pandemic with a focus on rituximab: Case reports from a single center experience in Australia

**DOI:** 10.3389/fmed.2023.1149742

**Published:** 2023-03-14

**Authors:** Ben Koszegi, Corey Stone, Dedee F. Murrell

**Affiliations:** ^1^Department of Dermatology, St George Hospital, Sydney, NSW, Australia; ^2^Faculty of Medicine, University of New South Wales, Sydney, NSW, Australia

**Keywords:** rituximab, COVID-19, pemphigus, immunosuppression, vaccine

## Abstract

Pemphigus is a rare group of autoimmune mucocutaneous blistering conditions for which the mainstay of treatment is immunosuppression. This is usually achieved with high dose corticosteroids as well as steroid sparing agents. Rituximab is now recommended as a first line treatment for moderate to severe pemphigus vulgaris, the commonest form of pemphigus, alongside corticosteroids. During the early stages of the COVID-19 pandemic the use of rituximab was reduced in our department due to its long term irreversible B-cell suppression. During the COVID-19 pandemic careful pharmacological selection was undertaken for our pemphigus patients to balance the risks of immunosuppression. To demonstrate this, we report three pemphigus patients who required treatment for COVID-19 and assessment throughout the pandemic. To date there has been limited published data regarding the clinical outcomes of pemphigus patients who have developed COVID-19 infections following rituximab infusions, especially in those patients who have received COVID-19 vaccinations. Following careful personalized consideration, all three pemphigus patients presented received rituximab infusions since the start of the COVID-19 pandemic. These patients had also received COVID-19 vaccinations prior to becoming infected with COVID-19. Each patient had a mild COVID-19 infection after receiving rituximab. We advocate for all pemphigus patients to have a full course of COVID-19 vaccinations. Antibody response to COVID-19 vaccinations should ideally be confirmed by measuring pemphigus patient’s SARS-CoV-2 antibodies prior to receiving rituximab.

## Introduction

Pemphigus is a rare group of autoimmune mucocutaneous blistering conditions, which are characterized by the production of autoantibodies against desmogleins (DSG) 1 and/or 3, leading to acantholysis (1). These conditions have a significant risk of mortality, especially if not managed effectively (1). The mainstay of treatment for pemphigus is immunosuppression which is achieved with high dose corticosteroids as well as steroid sparing agents. However, the use of these immunosuppressive agents must be balanced against increased infective risks in the context of the COVID-19 pandemic.

In 2018 the US Food and Drug Administration (FDA) approved rituximab for the management of adults with moderate to severe pemphigus vulgaris (PV). Rituximab is a monoclonal antibody which targets the CD20 antigen on B lymphocytes which results in suppression of CD20 expressing B lymphocytes, which leads to reduced plasma cells and therefore, reduced antibody production. This effect lasts approximately 6 months, with recovery in B lymphocytes expected 9–12 months following treatment. Rituximab is now recommended as a first line treatment for pemphigus in conjunction with high dose oral corticosteroids, Joly el al. (2) ultimately leading to higher remission rates and steroid sparing effects when compared to corticosteroid therapy alone (3). However, due to rituximab’s long lasting B-cell suppression, careful consideration has been given to its use for the management of pemphigus during the COVID-19 pandemic. Many clinicians elected to postpone rituximab infusions, especially in the early stages of the pandemic, prior to the availability of effective COVID-19 vaccinations (4, 5). Given the reduced use of rituximab, this prompted clinicians to pursue other treatment options for their pemphigus patients.

Highlighted by the three following PV cases, we report on our careful immunosuppressive pharmacologic selection and experience using rituximab during the COVID-19 pandemic. We also report on the outcomes of these PV patients who were subsequently diagnosed with COVID-19 after receiving rituximab infusions (Table 1). To our knowledge the following patients are the only three pemphigus patients known to our service who have contracted COVID-19 following at least one rituximab infusion since the start of the pandemic.

**TABLE 1:** Patient characteristics and timing of COVID-19 vaccinations, dates of rituximab (RTX) infusions and timing of COVID-19 infection in relation to most rituximab infusions.

Patient number	Age/sex	Dx	Comorbidities	COVID-19 vaccination history	SARS-CoV-2 antibodies	Date and dose of RTX infusions	Timing of COVID-19 infection post RTX^2^	Adjuvant treatments^3^	COVID-19 symptoms	Antiviral therapy^4^	Outcome
1	36/F	PV	Nil	1. Pfizer September 2021 2. Pfizer October 2021 3. Moderna May 2022	04/02/2022 IgG spike: detected Ab nucleocapsid: not detected^1^	10/01/2020: 500 mg 19/02/2020: 500 mg 04/03/2022: 500 mg	D20	Nil	Headache Fever Sore throat diarrhea Fatigue	No	Recovered
2	41/F	PV	Hypothyroidism	1. Pfizer July 2021 2. Pfizer August 2021 3. AstraZeneca October 2021 4. Moderna December 2021 5. Moderna January 2022	04/01/2022 IgG spike: detected Ab nucleocapsid: not detected^1^	07/2/2022: 1000 mg 21/2/2022: 1000 mg	D60	Weaning course of prednisone 5 mg	Cough Fatigue	Yes	Recovered
3	51/F	PV	Still’s disease	1. Pfizer September 2021 2. Pfizer October 2021 3. Moderna February 2022	03/11/2021 IgG spike: detected Ab nucleocapsid: not detected^1^	26/10/2020: 500 mg 12/10/2020: 500 mg 03/12/2021: 500 mg 18/11/2022: 500 mg	D90	TCS	Cough Nasal congestion Fatigue	Yes	Recovered

^1^Consistent with COVID-19 vaccination in an individual without previous COVID-19 infection. ^2^Interval in days (D) between rituximab infusion and COVID-19 infection. ^3^Pemphigus related adjuvant treatments taken during COVID-19 infection. ^4^COVID-19 antiviral therapy taken by patient during COVID-19 infection.

### Patient one

**FIGURE 1:**
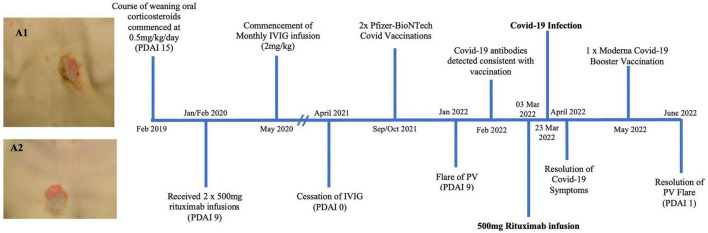
Timeline summary of patient one’s disease course (PDAI: pemphigus disease area index). Images A1 and A2 show erosions on the back (A2) and chest (A2) of patient one prior to receiving treatment detailed in this timeline.

A 36 year old female of Asian ethnicity presented in 2019 with a 2 year history of oral blisters, primarily affecting her cutaneous and mucosal buccal region (Figure 1). The diagnosis of PV was confirmed with histopathology, direct and indirect immunofluorescence. In 2019 her pemphigus disease area index (PDAI) on presentation was 15, indicating moderate disease activity. Her disease was initially controlled with a weaning course of prednisone 25 mg (0.5 mg/kg/day). However, upon weaning her prednisone to below 5 mg/day her disease severity worsened and she was prescribed two 500 mg rituximab infusion 2 weeks apart in early 2020. These initial rituximab infusions coincided with the onset of the COVID-19 pandemic, thus no further rituximab infusions were provided to the patient. In order to achieve disease control and reduce her prednisone requirement the patient received regular monthly intravenous immunoglobulin (IVIG) infusions. On review in May 2021 the patient had achieved complete remission with a PDAI score of 0 and hence her monthly IVIG infusions were ceased. The patient then had her initial COVID-19 vaccinations consisting of two Pfizer-BioNTech mRNA vaccines in September and October 2021. On review in early 2022 the patient developed a flare of the PV with new recurrent oral lesions and persistent right cheek blisters (PDAI activity score of 7). Her SARS-CoV-2 antibodies demonstrated evidence of serological immunity from her previous vaccinations. A 500 mg rituximab infusion was administered to the patient in March 2022. A total of 20 days after this rituximab infusion the patient tested positive to COVID-19 on a polymerase chain reaction (PCR) test. The patient had mild symptoms of headache, fever, sore throat and diarrhea. She did not experience a flare of her PV. These symptoms lasted for 12 days, however, the fatigue affected the patient for 3 months. Following the resolution of this fatigue, the patient has had no long term sequelae of her COVID-19 infection.

### Patient two

**FIGURE 2:**
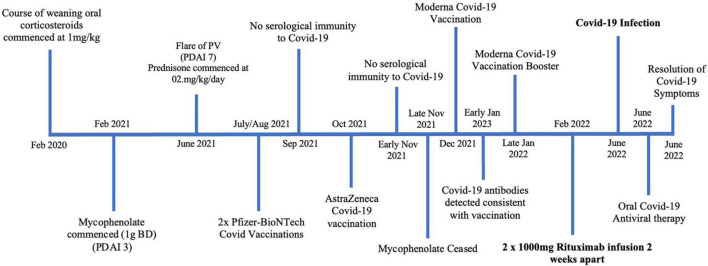
Timeline summary of patient two’s disease course.

A 41 year old female of Indian descent presented in February 2021 with a 1 year history of established PV, with lesions primarily affecting her oral mucosa, face and upper back (Figure 2). She had been previously managed on a course of prednisone 50 mg (1 mg/kg), which had been weaned to 20 mg/day by February 2021. At this initial review she demonstrated improved control of her disease, with only small and few oral lesions (PDAI activity score of 3). Accordingly her prednisone dose was weaned further and she was commenced on mycophenolate 1 g twice daily as a steroid sparing agent. However, in June 2021 upon weaning prednisone dose to 2 mg/day the patient had a flare of her PV, with a return of oral mucosal blisters (PDAI activity score of 7). Rituximab infusions were discussed with the patient, however, as she had not been vaccinated against COVID-19 this was not initiated. Her prednisone dose was increased to 7 mg/day and mycophenolate continued. The patient subsequently had a course of Pfizer-BioNTech COVID-19 vaccinations in July and August 2021, however, serological testing after 4 weeks confirmed that she did not have serological immunity. The patient was urgently referred to an immunologist who recommended that she receive an AstraZeneca COVID-19 vaccination booster which she received 4 weeks after her last Pfizer-BioNTech COVID-19 vaccination. However, again, her SARS-CoV-2 antibodies demonstrated no immunity 4 weeks following this booster vaccination. The patient’s mycophenolate was ceased and her prednisone dose was held at 7 mg/day. She then received two COVID-19 Moderna mRNA vaccinations in late December 2021. Her SARS-CoV-2 antibodies were measured 3 weeks following the first Moderna COVID-19 vaccine, which demonstrated serological immunity. Given this detected humoral response, the patient underwent two 1,000 mg rituximab infusions, 2 weeks apart, in February 2022. A total of 60 days after her last rituximab infusion, the patient tested positive to COVID-19 on a PCR test, however, she only had a mild disease course with a cough and fatigue which lasted 1 week. The patient was traveling overseas and received oral antiviral therapy. She has recovered from her COVID-19 infection with no long term sequelae.

### Patient three

**FIGURE 3:**
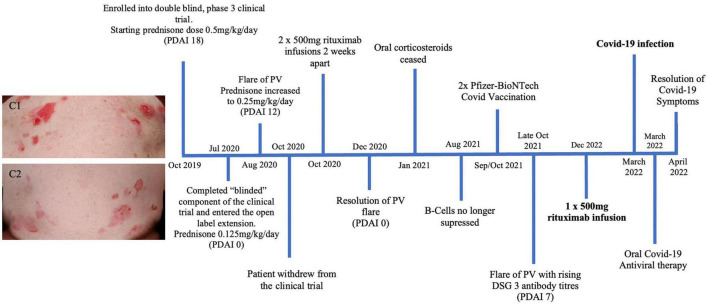
Timeline summary of patient three’s disease course. Images C1 and C2 show significant erosions on the upper and lower abdomen of patient three in 2019 prior to receiving treatment described in this timeline.

A 51 year old Caucasian female with a 7 year history of PV, was enrolled in a placebo controlled, phase 3 clinical trial for PV during October 2019 to October 2020 (Figure 3). Due to worsening PV disease control the patient decided to withdraw from the trial. The patient was taking 15 mg prednisone/day (0.25 mg/kg/day) at the time of exiting the clinical trial. Different management options were discussed with the patient, including increasing her prednisone and introducing steroid sparing agents including IVIG and rituximab. The patient wanted to avoid increasing her corticosteroid dose and lived in a rural location, making regular monthly IVIG infusions impractical. After careful consideration given the COVID-19 pandemic in late 2020, it was decided to proceed with rituximab infusions without increasing her prednisone dose. She received two 500 mg rituximab infusions 2 weeks apart in October 2020. Two months following the infusions she achieved clinical remission. Her prednisone was subsequently tapered and subsequently ceased over the following 2 months. The patient’s B-cell subtypes were measured monthly and the CD19 and CD20 B-cells were no longer suppressed as of August 2021. The patient’s COVID-19 vaccinations were delayed until this resolution of her B-cells. The patient subsequently had two Pfizer COVID-19 vaccinations in September/October 2021. From October 2021, the patient had worsening disease control with increasing DSG 3 antibody titre, necessitating a once off 500 mg rituximab infusion in December 2021. The patient then developed a COVID-19 infection 90 days following this infusion. Given her B-cell suppression, the patient received COVID-19 antiviral therapy. The patient reported mild symptoms of cough, nasal congestion and fatigue for 2 weeks. The patient recovered and had no long term sequelae from her COVID-19 infection.

## Discussion

The management of pemphigus presented many challenges during the COVID-19 pandemic. Pemphigus itself is associated with significant mortality if not managed effectively, however, the use of immunosuppressive treatment poses its own increased infective risk which has been exacerbated during the COVID-19 pandemic. The use of rituximab posed a particular challenge during the COVID-19 pandemic. The prolonged B-cell suppression produced by rituximab leads to a reduction in circulating pemphigus pathogenic autoantibodies, improving disease control. Its use has been shown to reduce the overall mortality in pemphigus and results in less adverse events compared to corticosteroid treatment monotherapy. However, given the prolonged B-cell suppression these patients have been shown to have increased COVID-19 related mortality (6, 7). It had been reported that patients with pemphigus or rheumatic diseases who were diagnosed with, and dying of, COVID-19 were more likely to be receiving rituximab (8–10). An Iranian retrospective cohort study by Professor Daneshpazhooh found that the risk of COVID-19 infection and hospitalization appears to decrease every month after the last rituximab infusion (8). While, a French study by Professor Joly found that patients with autoimmune blistering diseases (AIBD) and COVID-19 had a 5.9-fold higher risk of dying during the first wave of the COVID-19 pandemic in France than AIBD patients without COVID-19 (11). Moreover, the prolonged B-cell suppression is known to hamper effective humoral response to vaccinations by impeding the production of memory B-cells following antigen exposure (8). These considerations led to the avoidance of rituximab when possible to unvaccinated individuals during the pandemic which was recommended by an international group of experts (4).

These three cases discussed demonstrate the consideration of different management decisions during the COVID-19 pandemic. Australia adopted strict COVID-19 restrictions, including shutting international borders, restricting immigration and international travel and enforcing strict lockdowns. This kept case numbers low in comparison to Europe and the Americas until these restrictions were relaxed in late 2021 (12). However, due to the unpredictable nature of the pandemic we elected to, if possible, postpone rituximab infusions for our pemphigus patients until they were fully vaccinated and less virulent COVID-19 strains were present. As demonstrated in Case 1, other treatment options were explored with the patient including IVIG infusions. IVIG has been shown to be effective adjuvant treatment for both pemphigus and COVID-19 and hence was considered safe and effective to be given during the pandemic (4).

Other treatment options explored with patients in the initial stages of the pandemic included enrolling patients into pemphigus clinical trials including a new oral BTK inhibitor (ClinicalTrials.gov, identifier: NCT03762265). The benefit of this treatment is that it works *via* reversible covalent binding and therefore has a self-limited immunomodulatory effect (4). Given the self-limiting immunomodulatory effect, many pemphigus patients were enrolled into this trial during the pandemic as an alternative to rituximab infusions.

In our center we ensure that patients have serological immunity to COVID-19 vaccination by measuring SARS-CoV-2 serology. In the case of patient 2, despite an initial course of two Pfizer-BioNTech COVID-19 vaccinations, followed by an AstraZeneca COVID-19 booster vaccination, the patient had still not demonstrated a detectable humoral response to these vaccines. This necessitated the need to delay her rituximab infusions, until a humoral response to vaccination was demonstrated. Ensuring adequate COVID-19 vaccination has become standard practice at our center for all our pemphigus patients. We confirm humoral response to these vaccinations by measuring the patients SARS-CoV-2 antibody levels.

Of note, the three patients presented were not on high dose corticosteroids at the time of their COVID-19 infection. This was likely due to the steroid-sparing effects of their recent rituximab infusions. The patients and were also not taking any other steroid sparing agents at this time of infection. Patients 2 and 3 received oral COVID-19 antiviral therapy. These factors may have also contributed to their mild COVID-19 symptoms and disease course.

## Conclusion

We advocate for all pemphigus patients to have a full course of COVID-19 vaccinations ideally prior to patients receiving rituximab. This echoes the recommendation described by Pira et al. (13) in a recent review of COVID-19 and AIBD. The response to vaccination should ideally be confirmed by measuring the patient’s SARS-CoV-2 antibodies prior to receiving rituximab. Moreover, if no contraindications, we now recommend the use of COVID-19 antiviral therapy be provided to patients if they do become infected with COVID-19 and have had rituximab within the last 12 months, this is consistent with the Australian Government Department of Health Guidelines. This recommendation is communicated with patients and their primary care physicians. We have adopted this approach given the protracted nature of the COVID-19 pandemic. As discussed the three patients discussed are the only pemphigus patients who are known to our service to contract COVID-19. They all developed the infection likely to the milder, yet more infective, Omicron strain, despite a course of COVID-19 vaccinations with documented antibody response following vaccination. They all had mild COVID-19 symptoms, did not require hospitalization and had no long term sequelae.

## Data availability statement

The original contributions presented in this study are included in the article/supplementary material, further inquiries can be directed to the corresponding authors.

## Ethics statement

Written informed consent was obtained from the individual(s) for the publication of any potentially identifiable images or data included in this article.

## Author contributions

BK designed and wrote the first draft of the manuscript. DM supervised the work. All authors contributed to the preparation of the manuscript and approved the submitted version.
